# Supplementary material for: IGFs Potentiate TAC3-induced SLα Expression via Upregulation of TACR3 Expression in Grass Carp Pituitary Cells
Source: Cells. 2019 Aug 13;8(8):887. doi: 10.3390/cells8080887 (PMC6721824; doi:10.3390/cells8080887)
Supplement: Supplementary file 1 [file cells-08-00887-s001.zip › cells-562424-SI/Supplemental Figures.pdf]

Supplemental Figure 1

```
1          cacacacacacacacagacagacagacaga caggtg tcttct gagaga atc
52    ATGTCCTCATCAAGAAACTCCTCAAACTTCACC CATACC AACAGG TTCGCG CAGCCT CCA
       M  S  S  S  R  N  S  S  N  F  T  H  T  N  R  F  A  Q  P  P   20
112   TGGCGCGTCGCGCTCTGGGCGCTCGCGTACGCG CTGGTT ATGCTG GTGGCC GTGACG GGA
       W  R  V  A  L  W  A  L  A  Y  A  L  V  M  L  V  A  V  T  G   40
172   AACCTGGTCGTCATCTGGATCATCGTTGCGCAC AAGAGG ATGAGA ACCGTC ACCAAC TAC
       N  L  V  V  I  W  I  I  V  A  H  K  R  M  R  T  V  T  N  Y   60
232   TTTCTGCTCAACCTCGCGGTGTCTGACGTGTGC GTGGCC GCGCTC AACACG CTGGTG AAC
       F  L  L  N  L  A  V  S  D  V  C  V  A  A  L  N  T  L  V  N   80
292   TTCGTGTACGGCGTGCACGGGAACTGGTACTTC AGCAGC GCGTAC TGCCGC TTCCAC AAC
       F  V  Y  G  V  H  G  N  W  Y  F  S  S  A  Y  C  R  F  H  N  100
352   TTCTACCCGGTGGCCGCGGTGTTCGCGAGCATC TACTCC ATGAGC GCCATC GCCTTA GAC
       F  Y  P  V  A  A  V  F  A  S  I  Y  S  M  S  A  I  A  L  D  120
412   AGGTACATGGCGATCATCCACCCGATGAAGCCC CGCCTC TCAGCG ACGGTC ACCAAA GCT
       R  Y  M  A  I  I  H  P  M  K  P  R  L  S  A  T  V  T  K  A  140
472   GTGATCGCATGTGTGTGGATTCTGGCCGCATTT CTGGCC TTTCCA CTCTGC TTTTAC TCC
       V  I  A  C  V  W  I  L  A  A  F  L  A  F  P  L  C  F  Y  S  160
532   GTAACGGAAGTGAAGCAGCACAGGACGCTCTGC TATGTG GCCTGG CCGCGG CGCAAC GAC
       V  T  E  V  K  Q  H  R  T  L  C  Y  V  A  W  P  R  R  N  D  180
592   GACGCCTTCATATATCACGTGATCGTGGCGGTG CTGGTA TATCTC CTGCCT CTGGCA CTG
       D  A  F  I  Y  H  V  I  V  A  V  L  V  Y  L  L  P  L  A  L  200
652   ATGGCCGTCACCTACACCAGGGTGGGACTCGTG CTGTGG GGCGGT GGATTC CCCGGA CAC
       M  A  V  T  Y  T  R  V  G  L  V  L  W  G  G  G  F  P  G  H  220
712   TCCTCGGAAAACTTACAGGATCATCTGCAGGCC AAGAGA AAGGTT GTGAAG ATGATG GTG
       S  S  E  N  L  Q  D  H  L  Q  A  K  R  K  V  V  K  M  M  V  240
772   ATCGTGGTAGTAACATTTGCCATCTGCTGGCTC CCATAT CATGTG TATTTC ATCGCG ACG
       I  V  V  V  T  F  A  I  C  W  L  P  Y  H  V  Y  F  I  A  T  260
832   AGCTTCAACTGGAAGCTGACAAAATTCCAGTCG ATCCAG CAGGTG TATCTG TCGATC CTG
       S  F  N  W  K  L  T  K  F  Q  S  I  Q  Q  V  Y  L  S  I  L  280
892   TGGCTCTCCATGAGCTCCTCCATGTACAACCCC ATCATC TACTGC TGCCTC AACAGC AGG
       W  L  S  M  S  S  S  M  Y  N  P  I  I  Y  C  C  L  N  S  R  300
952   TTCCGCGCCGGCTTCAAGCGGGTGTTTCGCTGG TGTCCC TTCATT TATGTG TCGGAC TCT
       F  R  A  G  F  K  R  V  F  R  W  C  P  F  I  Y  V  S  D  S  320
1012  GATGAGATGGAACTCCAGACCACCCGATTCCAG CCAAAC CGCCAG AGCAGC CTGTAC ACG
       D  E  M  E  L  Q  T  T  R  F  Q  P  N  R  Q  S  S  L  Y  T  340
1072  GTCTCACGTGTGGAGTCCGACACCAACGCCAAC CCCGCC AGACGC AAGAGC TCCAGC ACC
       V  S  R  V  E  S  D  T  N  A  N  P  A  R  R  K  S  S  S  T  360
1132  AGCCACCGCAGCACCAGCCAATCACGACCCTCT GCCCGG CTGTCA CTCAAT GGTGGT CTC
       S  H  R  S  T  S  Q  S  R  P  S  A  R  L  S  L  N  G  G  L  380
1192  CATGGACACCCTACGAACGCCGCCGGTAATGAA CTCAGC TCAGAG TAGctc gcaaaa cac
       H  G  H  P  T  N  A  A  G  N  E  L  S  S  E  *               395
1252  taagctgcagaaatgacaggaaattcaccttca ggagct gctgaa tgccag cctcag tcc
1312  accagcttcactttgacctctctttcaattcca acatat ttaaaa ataact caccac aca
1372  tatgaaaatgaacacttctgaggacatacacac cggtct gttgac gaagat ttgtta aca
1432  ggttacactgtgtcctaaccagacacatcacaa taagat tccagc gacaag aagttt atc
1492  tgtccacaccagacgcgactacaagatgccaca aaatca atcgca aatcac agccaa tca
1552  taagagcatgtgggcggagtctctctgcaggcc cactac actcgc aaccta atcaat cca
1612  aaatcgctgcacgcactgcactcactaccaaat ggataa gcacaa aatcaa attcat aaa
1672  tatcacaccattttaacattattaaaaaaacgt actgag ttactg aaacaa tctttg tca
1732  tagaatacacttacttgttcacagcacgaaaaa aaaaaa aaaa
```

Supplemental Figure 1. Molecular cloning of grass carp TACR3. Nucleotide and deduced amino acid sequences of the grass carp TACR3. Numbering of the deduced amino acid sequences begins with the first methionine of the ORF to the right of each line. Nucleotide numbers are to the left of each line. The start and stop codons are shaded in gray. Predicted transmembrane domains (TMD1-TMD7) are boxed.

Supplemental Figure 2

	TMD1	TMD2	TMD3	TMD4	TMD5	TMD6	TMD7
Grass carp	100.0	100.0	100.0	100.0	100.0	100.0	100.0
Zebrafish	81.5	95.2	100.0	91.3	90.9	100.0	95.7
Frog	63.0	71.4	68.4	52.2	45.5	85.7	78.3
Bird	66.7	71.4	73.7	60.9	59.1	81.0	73.9
Mouse	70.4	76.2	73.7	60.9	59.1	95.2	82.6
Human	70.4	76.2	73.7	65.2	54.5	85.7	82.6

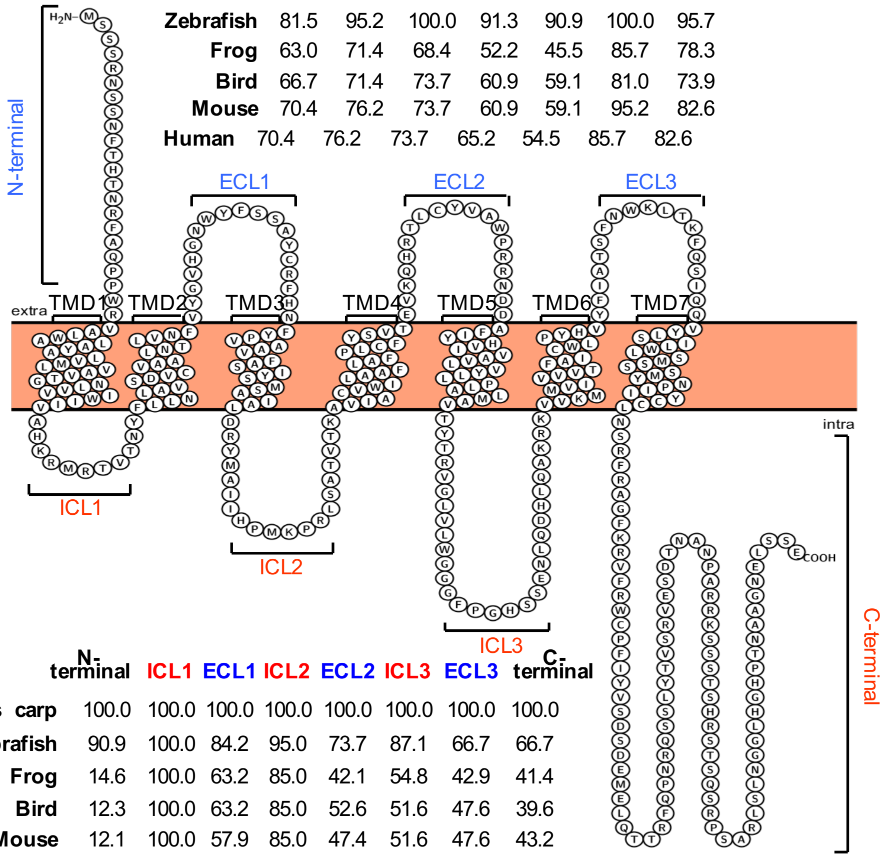

	N-terminal	ICL1	ECL1	ICL2	ECL2	ICL3	ECL3	C-terminal
Grass carp	100.0	100.0	100.0	100.0	100.0	100.0	100.0	100.0
Zebrafish	90.9	100.0	84.2	95.0	73.7	87.1	66.7	66.7
Frog	14.6	100.0	63.2	85.0	42.1	54.8	42.9	41.4
Bird	12.3	100.0	63.2	85.0	52.6	51.6	47.6	39.6
Mouse	12.1	100.0	57.9	85.0	47.4	51.6	47.6	43.2
Human	9.5	100.0	52.6	85.0	42.1	51.6	47.6	45.0

Supplemental Fig. 2. Sequence analysis of grass carp TACR3. Snake diagram of grass carp TACR3 with Protter program. The seven transmembrane domains, three intracellular domains and three extracellular domains are labeled as TMD1-7, ICL1-3 and ECL1-3, respectively. Sequence identities of transmembrane domains (TMD1-7) and extracellular domains (ECL1-3), intracellular domains (ICL1-3), N-terminal and C-terminal between grass carp and other vertebrates were showed in the table.
