# Supplementary material for: IGFs Potentiate TAC3-induced SLα Expression via Upregulation of TACR3 Expression in Grass Carp Pituitary Cells
Source: Cells. 2019 Aug 13;8(8):887. doi: 10.3390/cells8080887 (PMC6721824; doi:10.3390/cells8080887)
Supplement: Supplementary file 1 [file cells-08-00887-s001.zip › cells-562424-SI/Supplemental Table 1.pdf]

Supplemental Table 1

The information for the drugs used in receptor specificity and signal transduction.

Item	Function	Manufacture	Cat. No.
Hemokinin-1	NK1R agonist	Tocris Bioscience	1068
GR64349	NK2R agonist	Tocris Bioscience	1668
Senktide	NK3R agonist	Tocris Bioscience	1535
L-732128	NK1R antagonist	Tocris Bioscience	0868
GR159897	NK2R antagonist	Tocris Bioscience	1274
SB222200	NK3R antagonist	Tocris Bioscience	1393
8Br.cAMP	cAMP analog	Calbiochem	203800
Forskolin	AC activator	Sigma	F6886
Dic 8	DAG analog	Echelon	P-3008
TPA	PKC activator	Tocris Bioscience	1201
A23187	Ca^{2+} ionophore	Calbiochem	100105
Bay K8644	VSCC activator	Sigma	B112
MDL12330A	AC inhibitor	Merck	444200
H89	PKA inhibitor	Calbiochem	371963
GF109203X	PKC inhibitor	Sigma	B6292
U73122	PLC inactivator	Sigma	U6756
2-APB	IP3 receptor blocker	Calbiochem	100065
Nifedipine	VSCC blocker	Sigma	N-7634
Calmidazolium	CaM antagonist	RBI	C-100
KN62	CaMK-II blocker	Calbiochem	422706
PQ401	IGF-IR inhibitor	Calbiochem	407248
PPP	IGF-IR inhibitor	Calbiochem	407247
HNMPA	Insulin receptor inhibitor	Enzo	EI-248-0005
Wortmannin	PI_3K inhibitor	Calbiochem	681675
Rapamycin	mTOR inhibitor	Calbiochem	553210
PD98059	$MEK_{1/2}$ inhibitor	Calbiochem	513000
SB203580	$p_{38}MAPK$ inhibitor	Calbiochem	559389
