# Supplementary material for: IGFs Potentiate TAC3-induced SLα Expression via Upregulation of TACR3 Expression in Grass Carp Pituitary Cells
Source: Cells. 2019 Aug 13;8(8):887. doi: 10.3390/cells8080887 (PMC6721824; doi:10.3390/cells8080887)
Supplement: Supplementary file 1 [file cells-08-00887-s001.zip › cells-562424-SI/Supplemental Table 2. Antibody table.pdf]

Supplemental Table 2 Antibodies Used in Western Blot and Immunostaining

Peptide/protein target	Antigen sequence (if known)	Name of Antibody	Manufacturer, catalog #, and/or name of individual providing the antibody	Species raised in; monoclonal or polyclonal	RRID
**Phospho-MEK1/2 (Ser217/221)**	a synthetic phosphopeptide (KLP-coupled) corresponding to residues around Ser217/221 of human MEK1/2.	Phospho-MEK1/2 (Ser217/221) mAb	Cell Signaling Technology, Inc., catalog #9154	monoclonal IgG in Rabbit	AB_2138017
**MEK1/2**	a synthetic peptide (KLH coupled) covering the conserved region of human, rat and mouse MEK1/2.	MEK1/2 Antibody (for total MEK1/2)	Cell Signaling Technology, Inc., catalog #9122	polyclonal in Rabbit	AB_823567
**Activated (Diphosphorylated) ERK1/2**	a synthetic peptide (KLH coupled) with HTGFLTpEYpVAT sequence corresponding to the phosphorylated form of ERK-activation loop	Diphosphorylated ERK1/2 mAb	Sigma-Aldrich Co. , catalog #M8159	monoclonal IgG1 in Mouse	
**ERK-1/2**	a synthetic peptide (KLH coupled) with RRITVEEALAHPYLEQ YYDPTDE sequence derived from subdomain-XI of human ERK1/2.	ERK1/2 Antibody (for total ERK1/2)	Sigma-Aldrich Co. , catalog #M5670	polyclonal in Rabbit	
**Phospho-Akt (Ser473)**	a synthetic phosphopeptide (KLH-coupled) corresponding to residues surrounding Ser473 of mouse Akt.	Phospho-Akt (Ser473) Antibody	Cell Signaling Technology, Inc., catalog #9270S	polyclonal in Rabbit	AB_10694411
**Akt**	a synthetic peptide (KLH-coupled) derived from the carboxy-terminal sequence of mouse Akt.	Akt Antibody (for total Akt)	Cell Signaling Technology, Inc., catalog #9272	polyclonal in Rabbit	AB_329827
**Phospho-p_{38}MAPK (Thr180/Tyr182)**	a synthetic phosphopeptide (KLP-coupled) corresponding to residues surrounding Thr180/Tyr182 of human p38MAP kinase	Phospho-p_{38}MAPK (Thr180/Tyr182) Antibody	Cell Signaling Technology, catalog #5140	polyclonal in Rabbit	
**p_{38}MAPK**	a synthetic peptide (KLP-coupled) derived from human p_{38}MAP kinase	p_{38}MAPK Antibody (for total p_{38}MAPK)	MBL International, catalog # JM-3114-100	IgG in Rabbit	AB_592669
**β Actin**	a synthetic peptide (KLH coupled) covering the conserved region of human, rat and mouse Actin.	Anti-Actin mAB	Calbiochem, catalog #CP01	monoclonal IgM in Mouse	
**carp somatolactin alpha**	Recombinant carp SLα (GenBank No EF372074.1)	Antiserum against carp SLα	Prof AOL Wong, HKU, Hong Kong	polyclonal in Rabbit	AB_2631193
**carp somatolactin beta**	Recombinant carp SLβ (GenBank No EF372075.1)	Antiserum against carp SLβ	Prof AOL Wong, HKU, Hong Kong	polyclonal in Rabbit	AB_2631194

Abbreviations: KLH, keyhole limpet hemocyanin; mAb, monoclonal antibody.
