# Supplementary material for: IGFs Potentiate TAC3-induced SLα Expression via Upregulation of TACR3 Expression in Grass Carp Pituitary Cells
Source: Cells. 2019 Aug 13;8(8):887. doi: 10.3390/cells8080887 (PMC6721824; doi:10.3390/cells8080887)
Supplement: Supplementary file 1 [file cells-08-00887-s001.zip › cells-562424-SI/Supplemental Table 3.pdf]

Supplemental Table 3

Primers used for tissue distribution and quantitative real-time PCR.

Target	Forward primer	Reverse primer	Length	Annealing Tm	Accession No.
**Primers for tissue distribution**					
TACR3	GCCAAGAGAAAGGTTGTGAAGA	GTGTACATGCTGCTCTGGCG	330 bp	56℃	JQ254913
IGF-IR	ATCATGGAGCTGATGACACGA	CTCTGGAGACATCCAGCGCAC	312 bp	50℃	EU816193
β-Actin	CTGGTATCGTGATGGACTCT	AGCTCATAGCTCTTCTCCAG	280 bp	56℃	DQ211096
**qPCR primers**					
SLα	ACCCACTGTACTTCAATCTCC	CGTCGTAACGATCAAGAGTAG	283 bp	52℃	EF372074.1
TACR3	GCCAAGAGAAAGGTTGTGAAGA	GTGTACATGCTGCTCTGGCG	330 bp	56℃	JQ254913
β-Actin	CTGGTATCGTGATGGACTCT	AGCTCATAGCTCTTCTCCAG	280 bp	56℃	DQ211096
